# Coping strategies among Ethiopian migrant returnees who were in quarantine in the time of COVID-19: a center-based cross-sectional study

**DOI:** 10.1186/s40359-021-00699-z

**Published:** 2021-12-08

**Authors:** Yekoyealem Desie, Kassahun Habtamu, Mulat Asnake, Endirias Gina, Temesgen Mequanint

**Affiliations:** 1 School of Psychology, College of Education and Behavioral Studies, Addis Ababa University, P.O. Box: 150299, Addis Ababa, Ethiopia; 2 Research, Consultancy and Community Service Department, Ethiopian Police University, Sendafa, Ethiopia

**Keywords:** Coping strategies, COVID-19, Quarantine, Migrant returnees, Ethiopia

## Abstract

**Background:**

Following the coronavirus disease 2019 (COVID-19) outbreak, many numbers of Ethiopian migrant workers from the Middle East repatriated to their home country. Returnees who came back to Ethiopia during the early stages of COVID-19 went through difficult experiences of unplanned return and unfamiliar quarantine. Despite burgeoning studies on the coping strategies of the general population on stresses associated with the pandemic, there is lack of research on how returnees cope with challenges related to migration and quarantine experiences. The aim of this study was to examine the coping strategies used by returnees who were in mandatory quarantine in Addis Ababa, Ethiopia in the context of the COVID-19 pandemic.

**Methods:**

A center-based cross-sectional study was conducted with 405 migrant returnees who were in mandatory quarantine in Addis Ababa, Ethiopia. We developed a structured questionnaire to collect data about the socio-demographic, migration related, quarantine related and COVID-19 related characteristics of participants. We used the Brief COPE (Coping Orientation to Problems Experienced) scale to measure returnees coping strategies. Descriptive statistics and multiple regression analyses were used to determine extent of use of coping strategies and identify factors associated with them.

**Results:**

Emotion-focused coping mainly religious coping was the most frequently used coping strategy in the study group. Dysfunctional coping, however, was the least frequently employed coping strategy. Higher scores on emotion-focused and problem-focused coping strategies were associated with absence of perceived support from family and relatives after the quarantine and with no history of contact with COVID-19 suspected or infected person.

**Conclusions:**

The study shows that emotion-focused coping, particularly religious coping, was the most commonly used coping strategy among returnees who were in quarantine centers in the context of COVID-19. Returnees who perceived that they will not have support from family and relatives and those who were not exposed to the virus were more likely to use either emotion- or problem-focused coping strategies. Psychosocial reintegration efforts need to focus on enhancing returnees’ capacity to use adaptive coping strategies. We suggest in-depth qualitative studies for better understanding of returnees’ coping strategies and to facilitate reintegration activities.

**Supplementary Information:**

The online version contains supplementary material available at 10.1186/s40359-021-00699-z.

## Background

The coronavirus disease 2019 (COVID-19) pandemic is creating unparalleled public health and socioeconomic crisis worldwide. Compared to other infectious diseases and their respective burdens in recent human history, COVID-19 is bringing huge unanticipated threats to human well-being [1, 2]. One of the massive challenges associated with the pandemic is its negative mental health impacts. Review of studies on mental health and the outbreak of the pandemic have indicated that there was a significant upsurge in the prevalence of mental health problems linked with fear and stigma of the disease [3–5]. Data from China in the early months of the pandemic, for instance, showed that more than a quarter of the general population has experienced high level of psychological distress associated with the pandemic [6, 7]. Studies conducted in the United States also show a considerable rise in the incidence of mental health problems such as anxiety, depression, trauma and stress related disorders, substance use, and suicidal ideation accompanying the pandemic [8, 9]. Evidence emerges to show that the prevalence of mental health problems related to the uncertainty and tension brought about by the pandemic is growing in low and middle income countries [10–12].

The mental health impact of the COVID-19 pandemic is disproportionately high among vulnerable populations, particularly of migrant workers [13, 14]. On top of their pre-existing vulnerabilities of precarious living and working conditions, language and cultural barriers, repeated exposure to abuse and human rights violations, and limited access to mental healthcare services [15, 16], migrant workers are highly affected by the COVID-19 pandemic [13, 14, 17–19]. The common sources of stress among migrant workers associated with the pandemic include risk of infection of self and important others, deficiency of supplies, poor access to healthcare services, loss of job and income, and unfamiliar lockdown and quarantine experiences [20, 21]. A study in Kuwait showed that the pandemic has created additional life stressors to migrant workers [22]. Mukumbang et al. [12] also reported that COVID-19 has exacerbated the already existing susceptibilities of the migrant population in South Africa. The adverse mental health effects of COVID-19 may be worse for migrant workers who unexpectedly returned to their home country in the time of the pandemic.

Following the World Health Organization’s (WHO) declaration of the coronavirus outbreak as a global pandemic on March 11, 2020, significant numbers of Ethiopian migrant workers from the Middle East, mainly from Lebanon, United Arab Emirates, and Saudi Arabia are repatriated to their home country. Returnees who came back to Ethiopia during that time went through difficult experiences of unplanned return and novel quarantine experience. Many of them returned empty handed, as their incomes had been used for consumption and remittances to their families and in some cases, with unpaid salaries [23]. They are faced with multiple forms of economic and psychosocial problems. Several studies have documented that Ethiopian domestic workers in the Middle East, have huge vulnerabilities as they experience abuse and exploitation [24–29].

Upon their arrival, particularly in the first few months of the pandemic, migrant returnees were required to stay in mandatory 14-day government run quarantine facilities. Though quarantine has been taken as one effective way of managing the spread of COVID-19 [30], people who went through a mandatory quarantine may be exposed to mental health problems such as post-traumatic stress symptoms, confusion, anger, stigma, fear, and frustration [20, 31]. Studies from low and middle-income countries (LMICs) indicated that containment measures have tremendously affected the mental health of migrant workers [11, 12]. Overall, the unanticipated return of migrant workers coupled with their pre-existing vulnerabilities and tough quarantine experiences may create extra level of stress on returnees and this may lead them to develop severe forms of psychosocial problems. There have been efforts by the Ethiopian government and concerned non-governmental organizations (NGOs) to rehabilitate and reintegrate this group of returnees. Nevertheless, meaningful and successful rehabilitation and reintegration require an understanding of how returnees cope with life challenges.

When people are faced with stressful situations such as unplanned return and mandatory quarantine, they develop strategies that would help them manage impact of stressors. These responses to stressors are known as coping strategies [32]. Specifically, coping refers to the thought and behavioral strategies that an individual employs to deal with challenges that are appraised as taxing or go beyond their capacity [33]. Although coping responses differ across individuals and contexts, researchers clustered similar types of responses into three categories: problem-focused, emotion-focused, and dysfunctional or avoidance coping [34, 35]. While the problem-focused coping aims to manage threats by dealing directly with the source of the stress, the emotion-focused coping attempts to handle challenges by adapting one’s own response to the stressor. Dysfunctional coping, on the other hand, focuses on managing distress neither focusing on the source of the stress nor its emotional manifestations but through avoidance, venting of emotions, and behavioral and cognitive disengagement [34].

Several studies have been done on the coping strategies of migrants and refugees [36–41]. These studies showed that migrants’ coping vary depending on several factors like migration and acculturative experiences, status in the host country, and attachment to native culture. A study among Thai migrant workers in Norway indicated that migrants adopted the use of Thai cultural practices and Buddhist cognitive thinking as a prime coping strategy [41]. Ikafa and colleagues [36] also showed that African migrants in Australia relied on family support and faith in God as their coping strategies. Halcón et al. [42] further indicated that Somali and Ethiopian refugees most commonly used praying as a coping mechanism.

Few studies examined the coping strategies of Ethiopian migrant returnees [27, 28, 43]. Ethiopian women migrants to the Middle East use different coping strategies while they are working as housemaids in Arabian countries and after their homecoming [27]. Women migrants use problem-focused coping mainly escaping from employer’s house, and emotion-focused coping principally praying or crying to manage distress they face abroad, and sense-making and benefit-seeking to cope with traumatic experiences. According to Nisrane et al. [27] sense-making refers to returnees’ use of social comparison as a strategy to cope with stress. Benefit seeking, on the other hand, refers to returnees’ coping strategy either by interpreting their suffering as the price paid for the betterment of their families or understanding their challenging migration experience as a learning experience beneficial for future life. Despite mounting studies on the coping experiences of migrants and refugees, there is dearth of evidence on the coping strategies of returnees especially in the context of the COVID-19 pandemic.

There have been studies showing the psychological and coping responses towards emerging infectious disease outbreaks [44, 45]. Recent studies have also examined the way people responded to stress linked with COVID-19 [46]. Coping responses to stress associated with COVID-19 and quarantine varied across socio-economic and cultural contexts [47]. Despite burgeoning studies on coping strategies among the general population, studies that directly pertain to the coping strategies of returnees in the time of COVID-19 are lacking. The aim of this study was, therefore, to examine the coping strategies of Ethiopian migrant returnees to stressors associated with their migration and quarantine experiences in the context of the COVID-19 pandemic.

## Methods

### Study design

A center-based cross-sectional survey was conducted among migrant returnees who were in mandatory quarantine in Addis Ababa, Ethiopia in the context of COVID-19. The study aimed at identifying common coping strategies migrant returnees employ and factors associated with them. The study was conducted from 1st May to 15 June 2020.

### Study context and setting

The first case of the COVID-19 outbreak in Ethiopia was announced on March 13, 2020, two days after the WHO officially declared the disease as a global pandemic. The Federal Ministry of Health (MoH) proactively organized the COVID-19 Public Health Emergency Operations Center (PHEOC) on January 27 under the supervision of the Ethiopian Public Health Institute (EPHI). Over the following months, the MoH and EPHI worked in tandem to educate the public about the pandemic and implement firm precautionary measures to contain the spread of the virus.

Among the preventive measures taken were closing of schools and universities, the shutting down of night clubs and similar entertainment centers, the prohibition of sporting, religious and similar public gatherings, the closing of all international borders, the suspension of Ethiopian Airlines flights to over eighty destinations, and the imposition of a mandatory fourteen-day quarantine for all incoming international passengers. In addition, the government postponed the general election initially scheduled for August 2020. In order to further strengthen the protective measures, the government took more robust actions in the following months with the proclamation on April 8 of a state of emergency for a period of five months and imposed restrictions including a ban on meetings of more than four people, a reduction of passenger numbers on public transport vehicles by fifty percent, and the mandatory wearing of face masks in public places. These preventive measures were strictly implemented particularly in the early phase of the pandemic.

However, the concerted effort by the government to prevent the spread of COVID-19 was seriously challenged by the unexpected repatriation of many numbers of Ethiopian migrant workers from several Middle East countries. The Government was seriously challenged in ensuring a safe environment to receive the returnees and to support their psychosocial and economic rehabilitation and reintegration. As a preventive measure, the government arranged COVID-19 quarantine centers and requires all returnees to stay for 14 days with their expenses covered. The quarantine centers were established in hospitals, primary healthcare centers, schools, university campuses and convention centers. The current study was conducted in quarantine centers established in university campuses in Addis Ababa. The centers were student dormitories with basic facilities such as a separate room for each returnee, bed, blanket, and shared toilet and shower. Security forces were overseeing to make sure that no one is contacting with another in the center. Returnees were not allowed to go out and have physical contact with others in the center.

### Participants and sampling

We conducted the study in seven conveniently sampled quarantine centers established in three university campuses in Addis Ababa. We selected five centers (the Main Campus, College of Business and Economics Campus, College of Natural Sciences Campus, Lideta Campus, and Technology Campus) from Addis Ababa University, one center from Addis Ababa Science and Technology University, and another center from Ethiopian Civil Service University. In these seven quarantine centers there were about 6500 migrant returnees during the time of this study (2850 in the different quarantine centers in Addis Ababa University, 3060 in Addis Ababa Science and Technology University Center, and 590 in Ethiopian Civil Service University Center).

We approached 416 migrant returnees to take part in the study (182 from Addis Ababa University Centers, 38 from Ethiopian Civil Service University Center and 196 from Addis Ababa Science and Technology University Center). The inclusion criteria were being an Ethiopian migrant returnee during the time of COVID-19, stayed in one of the seven quarantine centers for at least ten days, being an adult (age 18 years or older), able to answer the survey questions in Amharic and able to give verbal informed consent. We retrieved 405 questionnaires yielding a response rate of 97.4%.

### Measures

#### Socio-demographic, quarantine, COVID-19 and migration related characteristics

We developed a structured questionnaire to collect data about the socio-demographic, migration related, quarantine related, and COVID-19 related characteristics of participants (see Additional file 1). The questionnaire consisted of 22 closed-ended items related to returnees’ socio-demographic characteristics (4 items), migration experiences (5 items), quarantine experiences (10 items) and COVID-19 related characteristics (3 items). The questionnaire was reviewed by experts who have experience on questionnaire development and scale adaption and those who have research experience on migration and health. We pilot tested the questionnaire with respondents having similar attributes as the main study participants. Based on the findings of the pilot study, we amended questions that were less understandable, sensitive and less acceptable.

### Coping strategies

We used the Brief COPE Scale [48] to measure migrant returnees coping strategies. The Brief-COPE is a 28-item abridged form of the full COPE (Coping Orientation to Problems Experienced) scale that is designed to measure the extent to which individuals respond to a broad range of stressors. It consists of 14 specific strategies, with two items each. The 14 strategies can be grouped into three higher order coping strategies: problem-focused, emotion-focused, and dysfunctional coping [48]. The problem focused coping contains strategies of active coping, instrumental support, and planning. The emotion focused coping includes strategies of acceptance, emotional social support, humor, positive reframing, and religion. The dysfunctional coping, on the other hand, consists of cognitive and behavioral disengagement, denial, self-distraction, self-blaming, and substance use and venting strategies [34].

The brief COPE has been used in various cultural contexts with diverse participants and demonstrated sound psychometric properties [49–54]. It has also been used in LMIC contexts, including in Ethiopia [28, 55]. The Brief COPE has been validated in Ethiopia in two studies among women with postpartum depression symptoms in rural Ethiopia [55] and among women labor migrant returnees from the Middle East countries [28]. In both of these studies, the COPE was reported to be valid and reliable. Confirmatory factor analysis has supported the three dimensions of coping (problem-focused, emotion-focused, and dysfunctional) [55].

In the current study, the brief COPE is translated into Amharic language, the official language in Ethiopia, by four members of the research team, who are fluent Amharic speakers and trained at masters’ or PhD degree level, following standard procedures [56, 57]. Senior members of the research team, who have training and experience in scale adaptation and validation, evaluated the relevance, cultural equivalence, acceptability and clarity of each item of the Amharic version of the scale. Participants rate each item on a Likert scale, ranging from 0 “I haven’t been doing this at all” to 3 “I’ve been doing this a lot.” Sample items from the scale include: ‘Thinking hard about what steps to take’ for problem-focused coping, ‘Trying to find comfort in my religion or spiritual beliefs’ for emotion-focused coping, and ‘Doing something to think about it less, such as watching TV, reading, daydreaming, or sleeping’ for dysfunctional coping. For this study, the internal consistency reliability coefficients for problem-focused, emotion-focused, and dysfunctional coping sub-scales were 0.74, 0.70, and 0.71, respectively.

### Data collection procedure

The process of data collection was executed by two masters level trained and experienced members of the research team. These members of the research team were also in charge of coordinating and supervising the quarantine centers employed by the Ethiopian Federal Ministry of Peace and Ministry of Health. The other three senior members of the research team supervised and coordinated the data collection process. The senior members of the research team trained those who executed the data collection, oversee participant recruitment and data collection and involve in checking and controlling data quality. A half-day orientation was delivered for those who executed the data collection on the purpose of the study, the contents of the data collection instruments, ethical matters, and on how to recruit and approach participants.

Data collection was carried out in quarantine centers (house-to-house) where migrant returnees were available via the guidance of key informants. Data collectors provided the questionnaire to those who gave consent and collected back the completed questionnaires after three days. For participants who were not literate, we administered the questionnaire in a face-to-face interview format. The senior members of the research team closely followed-up the data collection process.

### Data management and analysis

We entered and analyzed the data using the IBM Statistical Packages for the Social Sciences (SPSS) version 24 software. Data were checked for completeness and consistency before analysis began. We then conducted both descriptive and inferential statistical analyses to address the research questions. We used descriptive statistics to summarize the socio-demographic and other pertinent characteristics of the participants and determine the extent of use of coping strategies by migrant returnees.

We carried out simple and multiple regression analyses to examine the association of socio-demographic, migration related, quarantine related and COVID-19 related characteristics with migrants’ coping strategies (problem-focused, emotion-focused, and dysfunctional), separately. Factors that were associated with the outcome variables in the univariate models with *P *value < 0.2 were included in the corresponding multivariable models in order to limit the potential risk of over adjusting without compromising identification of potential predictors for the outcome variables. Standardized regression coefficients (β) (both crude and adjusted), with the corresponding 95% confidence interval, were used to estimate the strength of association between potential associated factors and the outcome variables. All statistical tests were set at α = 0.05 for significance.

### Ethical considerations

The study protocol was reviewed and approved by a committee established by the Office of the Vice President for Research and Technology Transfer (VPRTT) at Addis Ababa University. We secured a support letter from the VPRTT to collect data from the quarantine centers. We obtained permission to collect data from the coordinators of the quarantine centers by presenting a cooperation letter written from Addis Ababa University. Participation was voluntary and verbal informed consent was obtained from all the participants after the nature of the study was fully explained to them. We preferred verbal informed consent to written informed consent just to put respondents at ease since informants may not be comfortable to put their signature on paper in the Ethiopian socio-cultural context. Respondents were informed that they could withdraw at any time from the study and cease to respond to any question they felt uncomfortable. Information obtained from all the participants was anonymized and confidentiality was assured throughout the data collection process. Data collectors and field coordinators were urged to stick to all of the COVID-19 preventive measures.

## Results

### Characteristics of study participants

A total of 405 migrant returnees, who were in mandatory quarantine in Addis Ababa, participated in the study. Majority of the participants, (92.3%) were women ranged in age from 18 to 44 years (*Mean* = 25.80 years, *SD* = 3.58). A little more than half, (55.3%) of the participants completed secondary school. More than two-thirds, (70.1%) were single and about a quarter, (22.7%) currently married. Slightly above one-third, (34.8%) of the participants were not on job in the host country before their return, and 18.3% of them migrated through brokers.

Majority of the participants (83.2%) believed that quarantine limited their activities and social interaction. About 44% reported fear of discrimination after the quarantine. The majority, (85.2%) had no sufficient amount of money for living and to startup their own business after the quarantine, and about two-thirds of the participants, (64%) did not have plan of what to do after the quarantine. The majority of the participants (84.2%) reported that they had adequate knowledge about COVID-19 (Table 1).

**Table 1:** Socio-demographic, migration, quarantine, and COVID-19 related characteristics of participants, (*n* = 405)

Characteristics	Frequency	%
*Socio-demographic characteristics*		
Gender		
Female	374	92.3
Male	31	7.7
Age (in years), Mean (SD), 25.8 (3.58)		
Education		
Cannot read and write	23	5.7
Primary school	125	30.9
Secondary school	224	55.3
Post-secondary	33	8.1
Marital status		
Never married	284	70.1
Currently married	92	22.7
Previously married	29	7.2
*Migration related characteristics*		
Status in the host country before return		
On job	264	65.2
Detention center	66	16.3
Prison	43	10.6
Unemployed	32	7.9
Host (destination) country		
Jordan	293	72.3
United Arab Emirates	49	12.1
Qatar	26	6.4
Iran	12	3.0
Bahrain	8	2.0
Saudi Arabia	2	0.5
Ukraine	8	2.0
Australia	4	1.0
Spain	3	0.7
How did you go to the destination country? (way of migration)		
Through travel agency	331	81.7
Through broker	74	18.3
Underlying physical health problem		
Yes	20	4.9
No	385	95.1
Underlying mental health problem		
Yes	8	2.0
No	397	98.0
*Quarantine related characteristics*		
Fear of infection in quarantine center		
Yes, I was afraid	159	39.3
No, I was not afraid	246	60.7
Staying in quarantine protected me not to transmit the virus to family and community		
Yes	361	89.1
No	44	10.9
Quarantine limited my activities and social interaction		
Yes	337	83.2
No	68	16.8
Overall, services in the quarantine center was satisfactory		
Yes	216	53.3
No	189	46.7
I know the reason why I am here in quarantine		
Yes, I know	392	96.8
No, I don’t	13	3.2
I got sufficient information about the quarantine from the concerned body		
Yes	321	79.3
No	84	20.7
Fear of discrimination after the quarantine		
Yes	177	43.7
No	228	56.3
I can get support from family and relatives after the quarantine		
Yes, I can	245	60.5
No, I cannot	160	39.5
I have a plan of what to do after the quarantine		
Yes	146	36.0
No	259	64.0
I have sufficient amount of money for my living and startup business after the quarantine		
Yes	60	14.8
No	345	85.2
*COVID-19 related characteristics*		
I have adequate knowledge about the mode of transmission and prevention of the Coronavirus		
Yes, I have	341	84.2
No, I have not	64	15.8
I experienced headache, sore throat, breathing difficulty during my stay in quarantine		
Yes	15	3.7
No	390	96.3
I had contact with a COVID 19 suspected or infected person before the quarantine or I was exposed to situations before the quarantine		
Yes, I had	10	2.5
No, I had not	395	97.5
*Mental health related*		
Depression (n = 404)		
Normal (0–4)	182	45.0
Mild (5–6)	68	16.8
Moderate (7–10)	65	16.0
Severe (11–13)	41	10.1
Extremely severe (≥ 14)	48	11.9
Anxiety (n = 405)		
Normal (0–3)	207	51.1
Mild (4–5)	65	16.0
Moderate (6–7)	27	6.7
Severe (8–9)	35	8.6
Extremely severe (≥ 10)	71	17.5
Stress (n = 405)		
Normal (0–7)	259	64.4
Mild (8–9)	42	10.4
Moderate (10–12)	47	11.7
Severe (13–16)	29	7.2
Extremely severe (≥ 17)	25	6.2

*SD* standard deviation

As regards participants’ mental health characteristics, more than half of the returnees (55%) had depressive symptoms of which 22.0% had severe (10.1%) or extremely severe (11.9%) and 32.8% had mild (16.8%) or moderate (16.0%) symptoms of depression. About half (48.9%) of the participants had anxiety symptoms of which 26.1% had severe (8.6%) or extremely severe (17.5%) and 22.7% had mild (16.0%) or moderate (6.7%) symptoms of anxiety. One third (35.6%) of the returnees experienced symptoms of stress. Of them, 13.4% experienced severe (7.2%) or extremely severe (6.2%) stress, whereas 22.1% experienced mild (10.4%) or moderate (11.7%) stress (Table 1).

### Extent of use of coping strategies

Emotion-focused coping was the most frequently employed coping strategy among migrant returnees in quarantine centers. Particularly, religious coping: “trying to find comfort in my religion or spiritual beliefs” and “praying or meditating” were reported to be used ‘a lot’ by 73.3% and 69.9% of the participants, respectively. Problem-focused coping was also indicated as a commonly used coping strategy among returnees. The two items measuring the planning dimension of problem-solving coping: “Thinking hard about what steps to take” and “Trying to come up with a strategy about what to do” were reported to be used ‘a lot’ by 50.6% and 47.2% of the respondents, respectively. Dysfunctional coping especially substance use and self-blame were the least frequently used coping mechanisms among returnees. The uses of “alcohol or other drugs” and “self-criticism” were reported to be used ‘a lot’ by only 4.9% and 14.3% of the participants, respectively. However, the study further indicated that the self-distraction dimension of dysfunctional coping was another commonly used strategy reported by the returnees. The items assessing this dimension: “Doing something to think about it less, such as watching TV, reading, daydreaming, or sleeping” and “Turning to work or other activities to take my mind off things” were reported to be used ‘a lot’ by 49.4% and 31.9% of the respondents, respectively. The mean for the emotion-focused coping sub-scale was 27.28 (*SD* = 5.06) out of the possible 40. The mean for the problem-focused and dysfunctional coping strategies were 19.47 (*SD* = 4.60) and 27.51 (*SD* = 6.31) out of the possible 24 and 48, respectively (Table 2).

**Table 2:** Extent of use of coping strategies among migrant returnees in quarantine centers, (*n* = 405)

	Mean (SD)	Not at all	A little bit	A medium amount	A lot
Emotion focused coping strategies	22.28 (5.06)				
Trying to find comfort in my religion or spiritual beliefs	3.54 (.87)	23 (5.7)	33 (8.1)	52 (12.8)	297 (73.3)
Praying or meditating	3.46 (.92)	23 (5.7)	52 (12.8)	47 (11.6)	283 (69.9)
Looking for something good in what is happening	3.33 (.95)	22 (5.4)	70 (17.3)	65 (16)	248 (61.2)
Accepting the reality of the fact that it has happened	3.23 (.97)	30 (7.4)	64 (15.8)	95 (23.5)	216 (53.3)
Getting comfort and understanding from someone	3.12 (1.10)	49 (12.1)	63 (17)	72 (17.7)	215 (53.1)
Trying to see it in a different light to make it seem more positive	3.06 (1.10)	43 (10.6)	97 (24)	58 (14.3)	207 (51.1)
Learning to live with it	2.70 (1.10)	72 (17.8)	113 (27.9)	86 (21.2)	134 (33.1)
Making jokes about it	2.49 (1.12)	96 (23.7)	118 (29.1)	86 (21.2)	105 (25.9)
Making fun of the situation	2.36 (1.21)	139 (34.3)	89 (22)	68 (16.8)	109 (26.9)
Problem focused coping strategies	19.47 (4.60)				
Thinking hard about what steps to take	3.15 (1.01)	34 (8.4)	76 (18.5)	90 (22.2)	205 (50.6)
Trying to come up with a strategy about what to do	3.06 (1.05)	42 (10.4)	83 (20.5)	87 (21.5)	191 (47.2)
Trying to get advice or help from other people about what to do	2.93 (1.03)	40 (9.9)	108 (26.7)	98 (24.2)	159 (39.3)
Taking action to try to make the situation better	2.83 (1.03)	46 (11.4)	116 (28.6)	101 (24.9)	141 (34.8)
Getting help and advice from other people	2.75 (1.10)	63 (15.6)	118 (29.1)	80 (19.8)	144 (35.6)
Concentrating my efforts on doing something about the situation I'm in	2.72 (1.02)	51 (12.6)	129 31.9)	106 26.2)	119 (29.4)
Getting emotional support from others	2.05 (1.12)	172 (42.5)	110 (27.2)	52 (12.8)	70 (17.3)
Avoidance/dysfunctional coping strategies	27.51 (6.31)				
Doing something to think about it less, such as watching TV, reading, daydreaming, or sleeping	3.09 (1.02)	27 (6.7)	110 (27.2)	67 (16.5)	200 (49.4)
Turning to work or other activities to take my mind off things	2.80 (.97)	28 (6.9)	156 (38.5)	92 (22.7)	129 (31.9)
Expressing my negative feelings	2.78 (1.08)	64 (15.8)	98 (24.2)	104 (25.7)	138 (34.1)
Saying things to let my unpleasant feelings escape	2.76 (1.11)	67 (16.5)	107 (26.4)	88 (21.7)	143 (35.3)
Saying to myself "this isn't real	2.34 (1.11)	120 (29.6)	109 (26.9)	91 (22.5)	83 (20.5)
Giving up trying to deal with it	2.26 (1.02)	104 (25.7)	155 (38.3)	79 (19.5)	66 (16.3)
Giving up the attempt to cope	2.23 (1.14)	147 (36.3)	97 (24)	82 (20.2)	79 (19.5)
Refusing to believe that it has happened	2.24 (1.11)	138 (34.1)	108 (26.7)	83 (20.5)	76 (18.8)
Blaming myself for things that happened	1.98 (1.22)	223 (55.1)	52 (12.8)	47 (11.6)	83 (20.5)
Criticizing myself	1.87 (1.10)	216 (53.3)	83 (20.5)	47 (11.6)	58 (14.3)
Using alcohol or other drugs to help me get through it	1.78 (1.13)	253 (62.5)	48 (11.9)	43 (10.6)	60 (14.8)
Using alcohol or other drugs to make myself feel better	1.44 (.85)	300 (74.1)	50 (12.3)	35 (8.6)	20 (4.9)

*SD* standard deviation

### Factors associated with emotion-focused coping

In the unadjusted regression analyses, male gender, currently married marital status, have no fear of infection in quarantine centers, a belief that staying in quarantine does not protect me not to transmit the virus to family and community, absence of information about the quarantine, have no fear of discrimination after the quarantine, and lack of adequate knowledge about the transmission and prevention of the coronavirus were all negatively associated with emotion-focused coping. Migration through broker, perceived absence of support from family and relatives after the quarantine, and have no history of contact with a COVID-19 suspected or infected person were significantly associated with higher scores on the emotion-focused coping sub-scale. In the multivariable model, currently married (β = −0.23; CI − 3.89, − 1.62), have no fear of infection in quarantine centers (β = −0.17; CI  − 2.76, − 0.80), a belief that quarantine does not protect me not to transmit the virus to family and community (β = −0.13; CI − 3.7, − 0.68), have no fear of discrimination after the quarantine (β = −0.134; CI − 2.333, − 0.398), and lack of adequate knowledge about the transmission and prevention of the virus (β = −0.097; CI − 2.636, − 0.037) were negatively associated with emotion-focused coping. Absence of perceived support from family and relatives after the quarantine (β = 0.119; CI = 0.271, 2.182), and no history of contact with a COVID-19 suspected or infected person (β = 0.142; CI = 1.662, 7.601) were positively associated with emotion focused coping (Table 3).

**Table 3:** Factors associated with emotion-focused coping among migrant returnees (*n* = 405)

Characteristic	Mean (SD)	Crude β (95% CI)	Adjusted β (95% CI)
Age		0.10 (− 0.03, 0.24)	0.10 (− 0.01, .27)
Gender			
Female*	27.43 (4.98)		
Male	25.58 (5.67)	− **1.84 (**− **3.70, 0.01)**	− 0.07 (− 3.07, 0.59)
Education			
Not literate*	26.91 (5.624)		
Primary school	26.90 (5.069)	− 0.009 (− 2.27, 2.25)	
Secondary school	27.59 (5.025)	0.681 (− 1.50, 2.86)	
Post-secondary	26.88 (4.910)	− 0.034 (− 2.74, 2.67)	
Marital status			
Never married*	27.82 (4.85)		
Currently married	25.14 (5.23)	− **2.676 (**− **3.837,** − **1.514)**	− **0.23 (**− **3.89,** − **1.62)**
Previously married	28.86 (4.65)	1.045 (− 0.842, 2.932)	− 0.00 (− 1.87, 1.78)
Status in the host country			
On job*	27.28 (5.22)		
Detention center	27.48 (4.11)	0.21 (− 1.17, 1.58)	
Prison	26.93 (4.81)	− 0.35 (− 1.99, 1.29)	
Unemployed	28.76 (5.59)	0.09 (− 1.77, 1.96)	
How did you go to the destination country? (way of migration)			
Through travel agency*	27.05 (4.87)		
Through broker	28.35 (5.73)	**1.31 (0.03, 2.58)**	0.06 (− 0.41, 1.80)
Underlying physical health problem			
Yes*	28.30 (5.19)		
No	27.23 (5.05)	− 1.07 (− 3.35, 1.21)	
Underlying mental health problem			
Yes*	28.50 (4.93)		
No	27.26 (5.06)	− 1.24 (− 4.79, 2.31)	
Fear of infection in quarantine center			
Yes, I was afraid*	28.62 (5.6)		
No, I was not afraid	26.42 (4.46)	− **2.20 (**− **3.19,** − **1.22)**	− **0.17 (**− **2.76,** − **0.80)**
Staying in quarantine protected me not to transmit the virus to family and community			
Yes*	27.52 (4.94)		
No	25.39 (5.62)	− **2.13 (**− **3.70,** − **0.55)**	− **0.13 (**− **3.7,** − **0.68)**
Quarantine limited my activities and social interaction			
Yes*	27.42 (5.03)		
No	26.63 (5.17)	− 0.78 (− 2.10, 0.54)	
Overall, services in the quarantine center was satisfactory			
Yes*	27.32 (4.63)		
No	27.24 (5.51)	− 0.08 (− 1.07, 0.92)	
I know the reason why I am here in quarantine			
Yes, I know *	27.32 (5.02)		
No, I don’t	26.31 (6.13)	− 1.01 (− 3.81, 1.79)	
I got sufficient information about the quarantine from the concerned body			
Yes*	27.59 (4.79)		
No	26.12 (5.85)	− **1.470 (**− **2.681,** − **0.259)**	− 0.094 (− 2.372, 0.013)
Fear of discrimination after the quarantine			
Yes*	28.27 (5.18)		
No	26.52 (4.83)	− **1.754 (**− **2.766,** − **0.772)**	− **0.134 (**− **2.333,** − **0.398)**
I can get support from family and relatives after the quarantine			
Yes, I have*	26.67 (4.79)		
No, I have not	28.23 (5.32)	**1.566 (0.566, 2.566)**	**0.119 (0.271, 2.182)**
I have a plan of what to do after the quarantine			
Yes*	27.47 (4.695)		
No	27.18 (5.253)	− 0.284 (− 1.314, 0.745)	
I have sufficient amount of money for my living and startup business after the quarantine			
Yes*	27.08 (3.285)		
No	27.32 (5.306)	0.236 (− 1.156, 1.627)	
I have adequate knowledge about the mode of transmission and prevention of the Corona virus			
Yes, I have*	27.61 (5.02)		
No, I have not	25.55 (4.93)	− **2.063 (**− **3.403,** − **0.723)**	− **0.097 (**− **2.636,** − **0.037)**
I experienced headache, sore throat, breathing difficulty during my stay in quarantine			
Yes*	25.33 (4.05)		
No	27.36 (5.08)	2.026 (− 0.0585, 4.636)	− 0.019 (− 3.166, 2.14)
I had contact with a COVID 19 suspected or infected person or I was exposed to situations exposed before the quarantine			
Yes, I had*	23.10(4.58)		
No, I had not	27.39 (5.03)	**4.29 (1.132, 7.448)**	**0.142 (1.662, 7.601)**

Bold face indicates statistical significance at *p* < 0.05; * reference group

*SD* standard deviation, *CI* confidence interval

### Factors associated with problem-focused coping

A belief that staying in quarantine does not protect to transmit the virus to family and community, have no fear of discrimination after the quarantine, and have no a plan of what to do after the quarantine were negatively associated with problem-focused coping in both the univariate and multivariable models. Having sufficient amount of money for living and startup business after the quarantine (β = −0.11; CI − 2.66, − 0.06) was negatively associated with problem-focused coping in the adjusted model. In the univeriate analyses, perceived absence of support from family and relatives after the quarantine were negatively associated with higher scores on the problem-focused coping sub-scale. Absence of perceived support from family and relatives after the quarantine (β = 0.13; CI = 0.27, 2.09) and no history of contact with a COVID-19 suspected or infected person or no exposure to situations exposing to the virus (β = 0.10; 0.23, 5.91) were positively associated with problem focused-coping in the multivariable model (Table 4).

**Table 4:** Factors associated with problem-focused coping among migrant returnees (*n* = 405)

Characteristic	Mean (SD)	Crude β (95% CI)	Adjusted β (95% CI)
Age		0.05 (− 0.07, 0.17)	
Gender			
Female*	19.50 (4.54)		
Male	19.00 (5.32)	0.504 (− 2.19, 1.19)	
Education			
Not literate*	21.13 (4.39)		
Primary school	19.10 (4.76)	− 2.03 (− 4.08, 0.02)	
Secondary school	19.57 (4.52)	− 1.56 (− 3.54, 0.42)	
Post-secondary	19.00 (4.58)	− 2.13 (− 4.58, 0.32)	
Marital status			
Never married*	19.34 (4.46)		
Currently married	18.60 (4.81)	− 1.03 (− 2.12, 0.05)	
Previously married	20.54 (4.98)	0.90 (− 0.89, 2.69)	
Status in the host country			
On job*	19.48 ((4.59)		
Detention center	19.26 (4.84)	− 0.22 (− 1.47, 1.04)	
Prison	19.21 (4.77)	− 0.27 (− 1.77, 1.24)	
Unemployed	20.09 (4.79)	0.62 (− 1.08, 2.31)	
− 2.12, How did you go to the destination country? (way of migration)			
Through travel agency*	19.44 (4.63)		
Through broker	19.56 (4.50)	0.12 (− 1.05, 1.23)	
Underlying physical health problem			
Yes*	20.00 (4.17)		
No	19.44 (4.63)	− 0.56 (− 2.64, 1.51)	
Underlying mental health problem			
Yes*	21.38 (4.84)		
No	19.43 (4.59)	− 1.95 (− 5.18, 1.28)	
Fear of infection in quarantine center			
Yes, I was afraid*	19.81 (4.68)		
No, I was not afraid	19.25 (4.55)	− 0.56 (− 1.49, 0.36)	
Staying in quarantine protected me not to transmit the virus to family and community			
Yes*	19.74 (4.60)		
No	17.23 (3.95)	− **2.513 (**− **3.938,** − **1.087)**	− **0.18 (**− **4.092,** − **1.186)**
Quarantine limited my activities and social interaction			
Yes*	19.37 (4.65)		
No	19.94 (4.33)	0.57 (− 0.64, 1.78)	
Overall, services in the quarantine center was satisfactory			
Yes*	19.55 (4.59)		
No	19.37 (4.61)	− 0.18 (− 1.08, 0.73)	
I know the reason why I am here in quarantine			
Yes, I know*	19.53 (4.58)		
No, I don’t	17.38 (4.87)	− 2.15 (− 4.695, 0.394)	− 0.06 (− 4.15, 1.056)
I got sufficient information about the quarantine from the concerned body			
Yes*	19.63 (4.60)		
No	18.86 (4.59)	− .769 (− 1.877, .34)	− .0.03 (− 1.47, .80)
Fear of discrimination after the quarantine			
Yes*	20.15 (4.61)		
No	18.94 (4.53)	− **1.21 (**− **2.11, **− **.031)**	− **.139 (**− **2.21, **− **.37)**
I can get support from family and relatives after the quarantine			
Yes, I have*	19.09 (4.62)		
No, I have not	20.04 (4.53)	**0.95 (0.03, 1.87)**	**0.13 (0.27, 2.09)**
I have a plan of what to do after the quarantine			
Yes*	20.08 (4.59)		
No	19.12 (4.58)	− **0.97 (**− **1.90,** − **0.03)**	− **0.11 (**− **1.97,** − **0.07)**
I have sufficient amount of money for my living and startup business after the quarantine			
Yes*	20.28 (4.35)		
No	19.32 (4.64)	− 0.96 (− 2.23, 0.30)	− **0.11 (**− **2.66,** − **0.06)**
I have adequate knowledge about the mode of transmission and prevention of the Corona virus			
Yes, I have*	19.53 (4.63)		
No, I have not	19.13 (4.48)	− 0.41 (− 1.64, 0.83)	
I experienced headache, sore throat, breathing difficulty during my stay in quarantine			
Yes*	20.07 (3.67)		
No	19.44 (4.64)	− 0.63 (− 3.01, 1.76)	
I had contact with a COVID 19 suspected or infected person or I was exposed to situations exposed before the quarantine			
Yes, I had*	16.90 (4.33)		
No, I had not	19.53 (4.59)	2.63 (− 0.16, 5.52)	**0.10 (0.23, 5.91)**

Bold face indicates statistical significance at *p* < 0.05; * reference group

*SD* standard deviation, *CI* confidence interval

### Factors associated with dysfunctional coping

In the simple regression models, male gender and have no a plan of what to do after the quarantine were negatively associated with dysfunctional coping. Perceived absence of support from family and relatives after the quarantine, absence of adequate knowledge about the transmission and prevention of the coronavirus were significantly associated with increasing scores in the dysfunctional coping sub-scale. In the multivariable model, male gender (β = −0.14; CI − 5.78, 0.90), primary (β = −0.27; CI − 6.45, − 0.96) and secondary (β = −0.29; − 6.27, − 0.96) education levels, and have no a plan of what to do after the quarantine (β = −0.12; CI − 2.89, − 0.31) were negatively associated with dysfunctional coping (Table 5).

**Table 5:** Factors associated with dysfunctional coping among migrant returnees (*n* = 405)

Characteristic	Mean (SD)	Crude β (95% CI)	Adjusted β (95% CI)
Age		0.10 (− 0.16, 0.18)	
Gender			
Female*	27.78 (6.23)		
Male	24.27 (6.48)	− **3.51 (**− **5.84,** − **1.18)**	− **0.14 (**− **5.78, 0.90)**
Education			
Not literate	30.39 (7.04)		
Primary school	27.41 (6.28)	− 2.98 (− 5.79, − 0.17)	− **0.27(**− **6.45,** − **0.96)**
Secondary school	27.31 (5.89)	− 3.08 (− 5.79, − 0.37)	− **0.29 (**− **6.27,** − **0.96)**
Post-secondary	27.18 98.16)	− 3.21 (− 6.57, 0.15)	− 0.128 (− 6.15, 0.24)
Marital status			
Never married*	27.47 (6.28)		
Currently married	27.14 (6.58)	− 0.33 (− 1.82, 1.17)	
Previously married	29.14 (5.68)	1.67 (− 0.79, 4.13)	
Status in the host country			
On job	27.52 (6.33)		
Detention center	27.12 (6.21)	− 0.40 (− 2.11, 1.32)	
Prison	28.16 (5.64)	0.65 (− 1.40, 2.70)	
Unemployed	27.41 (6.31)	− 0.11 (− 2.44, 2.22)	
How did you go to the destination country? (way of migration)			
Through travel agency*	27.23 (6.01)		
Through broker	28.75 (7.41)	1.52 (− 0.082, 3.121)	0.043 (− 0.88, 2.27)
Underlying physical health problem			
Yes	28.65 (5.99)		
No	27.45 (6.33)	− 1.20 (− 4.05, 1.65)	
Underlying mental health problem			
Yes	28.25 (5.37)		
No	27.49 (6.33)	− 0.75 (− 5.19, 3.68)	
Fear of infection in quarantine center			
Yes, I was afraid*	27.24 (6.39)		
No, I was not afraid	27.69 (6.25)	0.45 (− 0.82, 1.73)	
Staying in quarantine protected me not to transmit the virus to family and community			
Yes*	27.68 (6.44)		
No	26.20 (5.06)	− 1.47 (− 3.45, − 0.51)	− 0.09 (− 3.75, − 0.16)
Quarantine limited my activities and social interaction			
Yes*	27.66 (6.51)		
No	26.79 (5.19)	− 0.87 (− 2.54, 0.80)	
Overall, services in the quarantine center was satisfactory			
Yes*	27.45 (6.26)		
No	27.58 (6.38)	− 0.13 (− 1.12, 1.38)	
I know the reason why I am here in quarantine			
Yes, I know	27.55 (6.32)		
No, I don’t	26.46 (5.20)	− 1.09 (− 4.59, 2.41)	
I got sufficient information about the quarantine from the concerned body			
Yes*	27.63 (6.40)		
No	27.05 (5.96)	− 0.59 (− 2.12, 0.95)	
Fear of discrimination after the quarantine			
Yes*	28.18 (6.36)		
No	26.99 (6.23)	− 1.19 (− 244, 0.06)	− 0.05 (− 1.93, 0.56)
I can get support from family and relatives after the quarantine			
Yes, I have*	27.01 (6.18)		
No, I have not	28.27 (6.45)	**1.26 (**− **0.01, 2.53)**	0.08 (− 0.24, 2.29)
I have a plan of what to do after the quarantine			
Yes*	28.38 (6.90)		
No	27.02 (5.91)	− **1.36 (**− **2.65,** − **0.07)**	− **0.12 (**− **2.89,** − **0.31)**
I have sufficient amount of money for my living and startup business after the quarantine			
Yes*	28.88 (6.25)		
No	27.28 (6.30)	− 1.60 (− 3.36, 0.16)	
I have adequate knowledge about the mode of transmission and prevention of the Corona virus			
Yes, I have*	27.19 (6.26)		
No, I have not	29.20 (6.33)	**2.02 (0.33, 3.70)**	0.08 (− 0.35, 3.14)
I experienced headache, sore throat, breathing difficulty during my stay in quarantine			
Yes*	29.80 (5.20)		
No	27.42 (6.34)	− 2.38 (− 5.64, 0.88)	0.03 (− 2.63, 4.27)
I had contact with a COVID 19 suspected or infected person or I was exposed to situations exposed before the quarantine			
Yes, I had*	25.50 (6.06)		
No, I had not	27.56 (6.31)	2.06 (− 1.91, 6.04)	

Bold face indicates statistical significance at *p* < 0.05; * reference group

*SD* standard deviation, *CI* confidence interval

## Discussion

In this center-based cross-sectional study, we found that migrant returnees most frequently employed emotion-focused coping over problem-focused and dysfunctional coping strategies. Religious coping, a type of emotion-focused coping strategy, was reported to be used ‘a lot’ by about three-fourth of the participants. About half of the respondents also indicated planning, a type of problem-solving coping, and self-distraction, a type of dysfunctional coping, as their coping strategies. Alcohol or use of other drugs and self-criticism, were the least frequently used coping strategies by migrant returnees in quarantine centers.

We also found that returnees use of emotion-focused coping was negatively associated with currently married, no fear of infection in quarantine centers, with returnees belief that staying in quarantine does not protect them not to transmit the virus to family and community, no fear of discrimination after the quarantine, and lack of adequate knowledge about the transmission and prevention of the virus. On the other hand, higher scores on emotion-focused and problem-focused coping strategies were associated with absence of perceived support from family and relatives after the quarantine and with no history of contact with COVID-19 suspected or infected person. Our findings also shows that men, returnees with primary and secondary education levels, and those who had no a plan of what to do after the quarantine were less likely to use dysfunctional coping.

Our finding about returnees’ frequent use of emotion-focused coping, particularly religious coping strategy, is consistent with a previous study on coping strategies of Ethiopian women labor migrant returnees from Middle East countries [28]. This study reported that women migrant returnees most commonly use emotion-focused coping strategies such as seeking emotional social support, religious coping, and positive reframing followed by problem-focused coping strategies of instrumental social support and active coping. Our finding is also in harmony with a study on the coping strategies of Somali and Ethiopian refugees [42]. This study indicated that praying is the primary source of coping among the refugees. The current finding is also congruent with the findings of a study on the coping strategies of women with postpartum depression in Ethiopia [55]. This study showed that emotion-focused coping strategy mainly religious coping was the most commonly employed coping strategy among women with post-partum depression in a rural Ethiopia setting.

Returnees’ frequent use of religious coping strategy to manage their stress associated with unanticipated return and novel tumultuous quarantine experience in the current study is with our expectation for different reasons. First, in situations like this where individuals do not have sufficient time to reflect on their sudden experiences and when they perceive that they have inadequate resource to cope with stress they tend to depend on religious coping [32, 35]. Religious coping is a mechanism by which individuals try to find comfort in their religion or spiritual beliefs through praying or meditating [35]. Second, the majority of the participants (92.3%) in the present study were women returnees. Several studies indicate that women more than men do use emotion-focused coping [58]. Lastly, the use of emotion-focused coping strategy more than the other types of coping strategies is expected in the Ethiopian socio-cultural situation where the majority identify themselves with their religion.

In this study, we also found that about half of the returnees relied on the use of problem-focused, mainly planning, coping strategy. This finding matches with our expectation that for those returnees who went through unexpected return, staying in quarantine for some time may provide them an opportunity to think about what measures to take and develop a plausible strategy of what to do for their life after the quarantine. In addition, returnees’ use of the self-distraction dimension of dysfunctional coping is also with our expectation. In situations like this where people are undergoing abrupt changes in their life they may develop behavioral patterns that temporarily help them to disengage from unpleasant thoughts associated with the problem. Dysfunctional coping, particularly alcohol or use of other drugs and self-criticism, are found to be the least frequently used coping strategy among the returnees. The low level use of alcohol or other drugs may be related to inaccessibility of alcoholic beverages and other drugs in mandatory quarantine centers. In addition, the low level use of self-criticism as a coping strategy may be related to the situation of their return. As participants of this study are forced returnees and passed through new quarantine experience which were beyond their control, returnees may attribute causes of their difficult experiences to external sources instead of taking all the blame for their own.

Returnees who perceived absence of support from family and relatives after the quarantine and with no history of contact with COVID-19 suspected or infected person were more likely to use both emotion- and problem-focused coping strategies. This indicates that returnees who anticipated that they will not have support from family and the community after the quarantine and those who were not exposed to the virus seem to cope with challenges either focusing on the source of the stress or managing emotions coming out of it. Our finding also show that men, returnees with primary and secondary education levels, and those who had no a plan of what to do after the quarantine were less likely to use dysfunctional coping.

As our knowledge goes, this may be the first study on coping strategies of migrant returnees in quarantine centers in the context of COVID-19. Findings from this study suggest important implications for intervention and research. Reintegration and rehabilitation intervention efforts in Ethiopia seem to narrowly focus on economic support of returnees, disregarding their mental health needs. However, studies among Ethiopian migrant returnees from the Middle East indicate that mental health is a major concern for the group [28, 43]. In addition, findings of the present study and another recent study by the same authors on the same group of participants [59] indicate a very high incidence of depression, anxiety and stress among the returnees associated with their migration, quarantine, and COVID-19 related experiences. Thus, the fragile nature of the mental health status of migrant returnees necessitates the need to prioritize the mental health challenges of returnees. One of the important elements in addressing the mental health needs of the returnees is the identification and strengthening of the positive coping strategies of the returnees. Strengthening the positive coping skills of returnees may help them not only to manage their prior life challenges but also serve as important life skills to deal with future challenges. Therefore, concerned government and nongovernmental agencies working on returnees’ reintegration and rehabilitation need to include the promotion of positive coping skills of returnees in their intervention efforts.

The present study has also implications for future research. As labor migration to the Middle East and return migration from the same region is a common phenomenon in Ethiopia, we strongly recommend prospective studies to qualitatively investigate the coping experiences of returnees for a more meaningful understanding and designing a comprehensive reintegration and rehabilitation intervention. However, our research has the following limitation. The study is a center-based study that included returnees who came back to Ethiopia mainly from Middle East countries and stayed in government arranged quarantine centers. Therefore, our sample is not representative in terms of capturing the experiences of returnees from other parts of the world and those who were in self-quarantine at home or in hotel quarantine settings.

## Conclusions

Our study showed that emotion-focused coping, particularly religious coping, is the most commonly used coping strategy among migrant returnees who were in quarantine centers in Addis Ababa, Ethiopia in the time of COVID-19. Migrants’ coping strategies were significantly associated with some socio-demographic, quarantine and COVID-19 related characteristics. Psychosocial rehabilitation efforts need to consider the development of returnees’ capacity to use more positive coping strategies. We suggest in-depth qualitative studies for a more meaningful understanding of coping strategies of migrant returnees in Ethiopia.

## Supplementary Information

**Additional file 1.** Socio-demographic, migration, quarantine and COVID-19 related characteristics questionnaire.

## Data Availability

The datasets used and/or analyzed during the current study are available from the corresponding author on reasonable request.

## Abbreviations

COVID-19: Coronavirus Disease 2019
EPHI: Ethiopian Public Health Institute
MoH: Federal Ministry of Health
PHEOC: Public Health Emergency Operations Center
NGO: Non-governmental organizations
COPE: Coping Orientation to Problems Experienced
WHO: World Health Organization
LMICs: Low and middle-income countries
SPSS: Statistical Packages for the Social Sciences
CI: Confidence interval
VPRTT: The Office of the Vice President for Research and Technology Transfer
SD: Standard deviation
